# Renalase Lowers Ambulatory Blood Pressure by Metabolizing Circulating Adrenaline

**DOI:** 10.1161/JAHA.112.002634

**Published:** 2012-08-24

**Authors:** Gary V. Desir, LieQi Tang, Peili Wang, Guoyong Li, Benedita Sampaio‐Maia, Janete Quelhas‐Santos, Manuel Pestana, Heino Velazquez

**Affiliations:** From Yale University School of Medicine, Department of Medicine, New Haven, CT (G.V.D., L.Q.T., P.W., G.L., H.V.); VA Connecticut Healthcare System (VACHS) Medical Center, West Haven, CT (G.V.D., L.Q.T., P.W., G.L., H.V.); Nephrology Research and Development Unit, Faculty of Medicine and Dental Medicine, University of Porto, Hospital S. João, Porto, Portugal (B.S.-M., J.Q.-S., M.P.)

**Keywords:** catecholamines, hypertension, kidney, norepinephrine

## Abstract

**Background:**

Blood pressure is acutely regulated by the sympathetic nervous system through the action of vasoactive hormones such as epinephrine, norepinephrine, and dopamine. Renalase, a recently described, secreted flavoprotein, acutely decreases systemic pressure when administered in vivo. Single‐nucleotide polymorphisms present in the gene are associated with hypertension, cardiac disease, and diabetes. Although renalase's crystal structure was recently solved, its natural substrate(s) remains undefined.

**Methods and Results:**

Using in vitro enzymatic assays and in vivo administration of recombinant renalase, we show that the protein functions as a flavin adenine dinucleotide– and nicotinamide adenine dinucleotide–dependent oxidase that lowers blood pressure by degrading plasma epinephrine. The enzyme also metabolizes the dopamine precursor l‐3,4‐dihydroxyphenylalanine but has low activity against dopamine and does not metabolize norepinephrine. To test if epinephrine and l‐3,4‐dihydroxyphenylalanine were renalase's only substrates, 17 246 unique small molecules were screened. Although the search revealed no additional, naturally occurring compounds, it identified dobutamine, isoproterenol, and α‐methyldopa as substrates of renalase. Mutational analysis was used to test if renalase's hypotensive effect correlated with its enzymatic activity. Single–amino acid mutations that decrease its enzymatic activity to varying degrees comparably reduce its hypotensive effect.

**Conclusions:**

Renalase metabolizes circulating epinephrine and l‐3,4‐dihydroxyphenylalanine, and its capacity to decrease blood pressure is directly correlated to its enzymatic activity. These findings highlight a previously unrecognized mechanism for epinephrine metabolism and blood pressure regulation, expand our understanding of the sympathetic nervous system, and could lead to the development of novel therapeutic modalities for the treatment of hypertension. **(*J Am Heart Assoc*. 2012;1:e002634 doi: 10.1161/JAHA.112.002634.)**

## Introduction

The catecholamines epinephrine, dopamine, and norepinephrine play a key role in the regulation of blood pressure (BP) and cardiovascular function through their action on central and peripheral adrenergic and dopaminergic receptors. The known pathway for the metabolism of these compounds involves uptake by neuronal and extraneuronal tissues and breakdown by the intracellular enzymes monoamine oxidase (MAO) A and B and catechol O methyl transferase. We recently identified renalase, a novel flavin adenine dinucleotide (FAD)–dependent oxidase that is secreted into blood by the kidney and is hypothesized to participate in catecholamine metabolism.^1,2^ Plasma renalase levels are decreased in animals subjected to subtotal nephrectomy (5/6 Nx) and in patients with chronic kidney disease and end‐stage renal disease. Examination of a global renalase‐knockout mouse model reveals that renalase deficiency is associated with hypertension that is most likely of neurogenic origin.^3^ Two single‐nucleotide polymorphisms in the renalase gene (rs2576178 GG genotype and rs2296545 CC) are associated with essential hypertension.^4^ The polymorphism rs2296545 CC results in a conservative amino acid change (glutamic to aspartic acid at amino acid 37) within the FAD‐binding domain and is associated with cardiac hypertrophy, ventricular dysfunction, poor exercise capacity, and inducible ischemia in persons with stable coronary artery disease.^5^ A significant reduction in cardiac renalase levels was observed in the 5/6 Nx rat model.^6,7^ This is particularly significant because the abnormal regulation of catecholamine metabolism contributes to the pathogenesis of left ventricular hypertrophy, ventricular arrhythmia, myocardial ischemia, and myocardial necrosis. The G allele of the renalase polymorphism rs10887800 appears to be associated with an increased incidence of stroke.^8^

Recent studies suggest that renalase could be involved in the pathogenesis of diabetes. A genome‐wide association study and meta‐analysis indicate that ≍42 loci affect the risk of type 1 diabetes.^9^ That study included 7514 cases of type 1 diabetes and 9045 reference controls, and 2 replication cohorts, 1 from Denmark and 1 from Great Britain. It confirmed linkage with most of the 24 previously identified loci, the strongest associations being with human leukocyte antigen (HLA), insulin (INS), protein tyrosine phosphatase, non receptor type 22 (lymphoid) (PTPN22), cytotoxic T lymphocyte associated protein 4 (CTLA4), and interleukin 2 receptor alpha (IL2RA). Moreover, it identified 18 novel loci, and the strongest evidence of association among these regions was achieved with the renalase gene (combined *P*=1.3×10^−28^). These findings were replicated in a southeast US white population.^10^ Renalase is expressed in the pancreas in insulin‐secreting cells, and the mechanisms that underlie its potential role in the development of type 1 diabetes have not been defined.

Although there is increasing evidence that renalase might be of relevance to the pathogenesis of common human diseases, and although its crystal structure has been solved,^11^ its mechanism of action remains undefined. The present work provides evidence that epinephrine and the catecholamine precursor l‐3,4‐dihydroxyphenylalanine (l‐DOPA) are renalase's physiological substrates.

## Methods

### Reagents

The following reagents were obtained from Sigma Aldrich (St. Louis, MO): reduced form of nicotinamide adenine dinucleotide (NADH) (#N4505), epinephrine hydrochloride (#E4642), reduced form of nicotinamide adenine dinucleotide phosphate (NADPH) (#N6505), catalase (#C40), resazurin (#R7017), superoxide dismutase (SOD) (#S7446), FAD (#F6625), and enalapril maleate (E 6888). The Amplex Red Monoamine Oxidase Assay Kit was obtained from Molecular Probes (#A12214) and contained the resorufin (#424455) used to construct the standard curves.

### Synthesis of Human Renalase1

The gene sequence of human renalase 1 (hRenalase1) was subjected to codon optimization to facilitate expression in *E coli*. Untagged, recombinant hRenalase1 (amino acids 1 to 342) was generated by cloning the coding region into the Ned1/XhoI sites of the pET27b+ vector (Novagen, Madison, WI). For some studies, amino acid mutations were made with a Quick Change Mutagenesis kit (Stratagene). *E coli* BL21 were transformed and grown at 37°C for 16 hours with 0.1 μmol/L FAD. Isopropyl β‐d‐1‐thiogalactopyranoside was added for the last 3.5 hours of culture. Recombinant renalase was purified from inclusion bodies and refolded by dilution in the presence of FAD. All refolding steps were carried out at 4°C. Inclusion bodies were dissolved in 10 mL of solubilization buffer (8 mol/L urea, 100 mmol/L Tris, pH 10.5, 1 mmol/L glycine, 100 mmol/L β‐mercaptoethanol) by stirring for 60 minutes. Insoluble material was removed by centrifugation at 12 000×*g* for 15 minutes, and the supernatant was passed through a 0.2‐μm filter. The protein solution was adjusted to a final absorbance (A_280_) value of 2 with solubilization buffer without β‐mercaptoethanol. The pH was adjusted to 10.5, and the refolding process was initiated by adding 20 volumes of cold refolding buffer (20 mmol/L Tris, pH 10.5, 10% glycerol, 10 mmol/L DTT, 50 μmol/L FAD) with continuous stirring. The mixture was incubated for 2 hours, and the pH was reduced from 10.5 to 8.2 over 72 hours by continuous addition of hydrochloric acid via an automatic peristaltic pump. Once the pH had reached the desired value, the protein solution was incubated for 2 days at 4°C. The refolded protein was concentrated ≍15‐fold with a centrifugal filter device, and precipitated material was removed by centrifugation at 12 000×*g* for 15 minutes. Buffer exchange was carried out by dialysis against 100 volumes of dialysis buffer (25 mmol/L Bis‐Tris, pH 6.5, 10% glycerol, 10 mmol/L NaCl, 1 mmol/L EDTA, 0.5 mmol/L DTT) for 24 hours at 4°C. Precipitated protein was removed by centrifugation at 12 000×*g* for 60 minutes, and the renalase was concentrated to a final concentration of 0.5 to 1 mg/mL and stored frozen at −80°C. Refolded hRenalase1 (0.1 mg/mL) was analyzed on a high‐pressure liquid chromatography (HPLC) molecular sizing column (Agilent 1100 series HPLC, Biorad Gel filtration [300 mm×7.8 mm] column, catalog #125‐0062) at a flow rate of 0.5 mL/min (25 mmol/L Tris HCl pH 7.5, 10 mmol/L NaCl, 1 mmol/L EDTA, 10% glycerol, 0.5 mmol/L DTT) and an inject volume of 40 μL. Simultaneous ultraviolet absorbance readings were acquired at 280 nm for protein detection and 450 nm for FAD detection.

### Measurement of Renalase's Enzymatic Activity

#### NADH Oxidase Assay

The assay buffer contained 25 mmol/L Tris, pH 7.5, and 5 mmol/L NaCl. NADH was made fresh and added to a final concentration ranging from 1 to 1000 μmol/L. The reactions were initiated by adding 4 to 20 μg of recombinant renalase to 200 μL of assay buffer in 96‐well plate cuvettes (0.6‐cm path‐length). Four to twenty micrograms of bovine serum albumin served as a negative control. Absorbance at 340 nm was measured in a plate reader at 37°C and recorded every 4 minutes for up to 60 minutes. The amount of NADH oxidized to nicotinamide adenine dinucleotide (NAD+) was calculated from the decrease in absorbance at 340 nm with a molar extinction coefficient of 6220 M^−1^ cm^−1^ at 340 nm. Background correction was achieved by subtracting the changes in absorbance obtained with bovine serum albumin. To estimate kinetic parameters (Michaelis‐Menten constant [substrate concentration at half maximal velocity {K_m_}] and maximal velocity [V_max_]), initial velocity was plotted against substrate concentration, and the data were fitted to the Michaelis‐Menten equation by nonlinear regression (GraphPad Prism, GraphPad Software, Inc).

#### HPLC With Electrochemical Detection

The enzymatic assay was carried out for 1 minute at 37°C in buffer containing dopamine, 25 mmol/L Tris, pH 7.5, 5 mmol/L NaCl, and 250 mmol/L NADH. The reaction was stopped by adding 2 mol/L perchloric acid in an amount equal to 10% of the final reaction volume. Dopamine levels were assayed by HPLC with electrochemical detection (HPLC‐ED) (lower limit of detection of ≍350 fmol), as previously described.^12^ Kinetic parameters (K_m_ and V_max_) were calculated by plotting substrate consumption against substrate concentration and fitting the data to the Michaelis‐Menten equation by nonlinear regression (GraphPad Prism, GraphPad Software, Inc).

#### Resazurin Reduction Assay

Resazurin, a nonfluorescent compound, can be reduced by oxidoreductases to generate resorufin, a fluorescent metabolite.^13^ The assay buffer (total volume of 200 μL) contained 25 mmol/L Tris, pH 7.5, 5 mmol/L NaCl, and 50 μmol/L resazurin. NADH concentration was varied from 0 to 2 mmol/L, and epinephrine concentration ranged from 0 to 1.6 mmol/L. Each reaction contained either recombinant renalase or bovine serum albumin. Fluorescence intensity was measured with a fluorescence plate reader (560‐nm excitation filter, 590‐nm emission filter) at 37°C and recorded every 2 minutes for up to 30 minutes. The amount of resorufin generated was determined by using a standard curve constructed with resorufin concentrations ranging from 0 to 40 μmol/L. Changes in fluorescence were linear up to a resorufin concentration of 3 μmol/L. Background correction was achieved by subtracting the changes in fluorescence obtained with bovine serum albumin.

### Small Molecule Screen

The following libraries (17 246 compounds) were screened at the Small Molecule Discovery Center at Yale University: MicroSourceGen‐Plus Collection (960 compounds, including small molecules with known bioactivity, and medicines currently in use), the NIH Clinical Collection (446 compounds used in human clinical trials), the MicroSource Natural Product Collection (800 compounds, including characterized alkaloids, flavanoids, sterols, terpenes, and stilbenes), and ChemBridge DIVERSet (15 040 compounds, a broad set of biologically relevant molecules).

Singleton 20‐μL reactions were run in 384‐well plates with NADH, hRenalase1 (2 μg/mL), and test compounds at 10 μmol/L. NADH oxidation to NAD+ was monitored through its coupling to resazurin (10 μmol/L) reduction to resorufin, which is accompanied by an increase in fluorescence at excitation 535 nm / emission 590 nm. Fluorescence emission at 590 nm (excitation 535 nm) was quantified on a PerkinElmer EnVision plate reader immediately after the initiation of the reaction (F_0_) and ≍1 hour after incubation at room temperature (F). Positive control wells lacked renalase and exhibited a low fluorescence signal change, whereas negative control wells lacked compound and exhibited a high fluorescence signal change. The difference in fluorescence signal (F–F_0_) was used to determine percent effect with the following equation for each well:

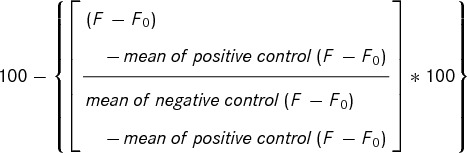


Signal‐to‐background ratios were calculated by:

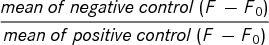


Z’ was calculated according the methods developed by Zhang and colleagues, ^14^ with the formula:

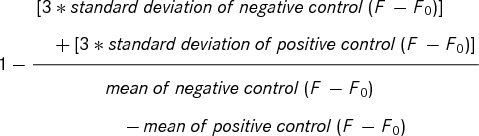


Thresholds for assay inhibition or activation were set by using statistics based on the distribution of percent effect values of sample wells. The threshold for assay inhibition was calculated as the median percent effect of the sample population plus 3 standard deviations, and the threshold for assay activation was set by the median percent effect of the sample population minus 3 standard deviations.

Compounds that increased resazurin reduction were flagged as potential substrates. These were rescreened for their ability to increase NADH oxidation by using the NADH oxidase activity assay described above.

### Ambulatory BP Monitoring and Drug Administration

All animal experimentations were conducted in accordance with the National Institutes of Health Guidelines for the Care and Use of Laboratory Animals.

#### Spontaneously Hypertensive Stroke‐Prone Rats

Animals weighing 275 g were obtained from Charles River Laboratories, were group housed, and initially were fed a low‐sodium diet (Teklad 7034, Harlan) and synchronized to a 12/12‐hour light (6 am to 6 pm) and dark (6 pm to 6 am) cycle. BP was monitored by surgically implanting a BP transducer (TA11PA‐C40, Data Sciences International, St. Paul, MN) that emits a radio signal monitored by telemetry into the aorta below the level of the renal arteries. Approximately 2 weeks were allowed for the animals to recover and for establishment of a baseline circadian rhythm. Hypertension was induced by switching to a high‐NaCl diet (Teklad 92012) for ≍4 weeks. BP was measured for 10 seconds every 5 minutes and recorded on a computer. Moving averages of BP every 1 hour were calculated, graphed in software provided by DSI, and also exported to a spreadsheet for analysis. Rats gained weight throughout the study.

#### Chronic Kidney Disease Model (5/6 Nx)

Male Sprague Dawley rats (200 to 300 g body weight) were fed a standard (24%) protein diet (Purina, St. Louis, MO) and were synchronized to a 12/12‐hour light (6 am to 6 pm) and dark (6 pm to 6 am) cycle. Subtotal nephrectomy was achieved by surgically removing the right kidney and ligating the arterial blood supply to two thirds of the left kidney. To monitor BP, a transducer (TA11PA‐C40, Data Sciences International, St. Paul, MN) that emits a radio signal was inserted into the aorta below the level of the renal arteries. Approximately 2 weeks were allowed for the animals to recover and for establishment of a baseline circadian rhythm. BP was measured for 10 seconds every 5 minutes and recorded, and moving averages of BP every 1 hour were calculated. Rats gained weight throughout the study.

Recombinant renalase (1.3 mg/kg) or vehicle (renalase dialysis buffer) was administered by a single subcutaneous injection in the flank or interscapular region. Enalapril (1 to 5 mg/kg per day) was added to the drinking water supply for 24 hours. The administered dose was calculated on the basis of total water consumption.

### Statistical Analysis

The Wilcoxon rank test and the Mann‐Whitney test were used for paired and unpaired data, respectively. When appropriate, nonparametric repeated‐measures ANOVA (Friedman test) was used to evaluate statistical significance. When the Friedman test revealed statistical significance, Dunn's test was used for pairwise comparisons. All data are mean ± standard error of the mean (mean±SEM), and values of *P*<0.05 were accepted as a statistically significant difference.

To estimate kinetic parameters (K_m_ and V_max_), initial velocity was plotted against substrate concentration, and the data were fitted (least‐squares fit) to the Michaelis‐Menten equation by nonlinear regression (GraphPad Prism, GraphPad Software, Inc). The initial K_m_ value was constrained to >0.

The authors had full access to and take full responsibility for the integrity of the data. All authors have read and agree to the manuscript as written.

## Results

### Enzymatic Activity of Recombinant hRenalase1

To facilitate expression in *E coli*, we designed a synthetic hRenalase1 gene, in which ≍30% of nucleotides were substituted to optimize codon usage and to remove putative translational pause signals while preserving the native amino acid sequence. Compared to the natural gene, transfection with the synthetic gene increased recombinant hRenalase1 expression by ≍200‐fold (Figure 1A). Ninety‐five percent of the synthesized protein was recovered as inclusion bodies, which were purified, denatured, and refolded by dilution. Size‐exclusion chromatography revealed that hRenalase1 elutes as a FAD‐bound monomer at ≍10.4 minutes, and a small amount of free FAD can be observed to elute at ≍13 minutes (Figure 1B). The homogeneity of the samples was evaluated by SDS‐PAGE under reducing and nonreducing conditions. Refolded renalase was >98% pure as assessed by Coomassie staining and migrated as a 38‐kDa band, a value identical to its predicted molecular mass (Figure 1C), which indicated that recombinant hRenalase1 predominantly exists in a monomeric form.

**Figure:**
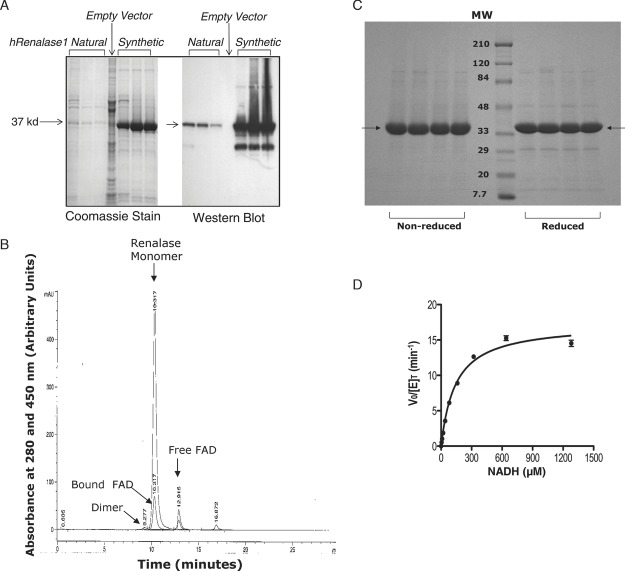
Effect of gene optimization and synthesis of active recombinant hRenalase1 in *E coli*. A, left, Crude *E coli* lysates separated by SDS‐PAGE and protein visualized by Coomassie staining. A, right, Western blot of crude *E coli* lysates with the use of an anti‐renalase antibody. Arrow indicates hRenalase1 protein; Natural, wild‐type hRenalase1 cDNA; Synthetic, codon‐optimized hRenalase1 cDNA; and Empty Vector, pET27b vector without hRenalase1. B, Elution profile of refolded recombinant renalase subjected to size‐exclusion chromatography. C, Comparison of recombinant renalase under reducing and nonreducing conditions; 10 μg refolded renalase loaded in each lane. MW indicates molecular weight markers; arrows, renalase bands. D, Steady‐state rate of NADH oxidation by hRenalase1 monitored by changes in absorbance in 25 mmol/L Tris, pH 7.5, and 5 mmol/L NaCl, at 37°C. V_0_ indicates initial velocity;. [E]T, renalase concentration.

We had reported previously that hRenalase1 could catalyze the metabolism of catecholamines.^1^ However, the measured turnover rates were low enough to cast doubt on the physiological relevance of renalase with regard to catecholamine metabolism.^15^ We later reported that NAD(P)H was required as a cofactor for the enzymatic function of renalase,^5^ a finding subsequently confirmed by others.^11^ The enzymatic activity of hRenalase1 was assessed by monitoring the conversion of NADH to NAD+ and measuring the resulting change in absorbance at 340 nm. The steady‐state kinetic data for NADH oxidation are plotted in Figure 1D, and the parameters for NADH and NADPH are shown in Table 1. These data indicate that hRenalase1 possesses significant NADH and NADPH oxidase activity (Equation 1) and has higher specificity for NADH.

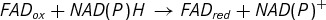
(1)
where FAD_ox_ is the oxidized form of FAD, and FAD_red_ is the reduced form of FAD.

**Table 1.** Parameters for NADH and NADPH

Substrate	k_cat_, min^−1^	K_m_, μmol/L	k_cat_/K_m_, min^−1^ μmol/L^−1^
NADH	17.35±0.85	137.80±21.91	0.13
NADPH	7.01±1.18	1400.23±381.1	0.005

NADH markedly increases the rate of epinephrine metabolism (Figure 2A; turnover rate [k_cat_] = 66.46 and 0.09 min^−1^ with and without NADH, respectively). In the presence of NADH, hRenalase1 also metabolizes l‐DOPA (k_cat_=42.32 min^−1^) and is minimally active against dopamine (k_cat_=0.11 min^−1^; kinetic parameters determined by HPLC‐ED) but has no effect on norepinephrine, serotonin, and tryptamine (Figure 2B and 2C).

**Figure:**
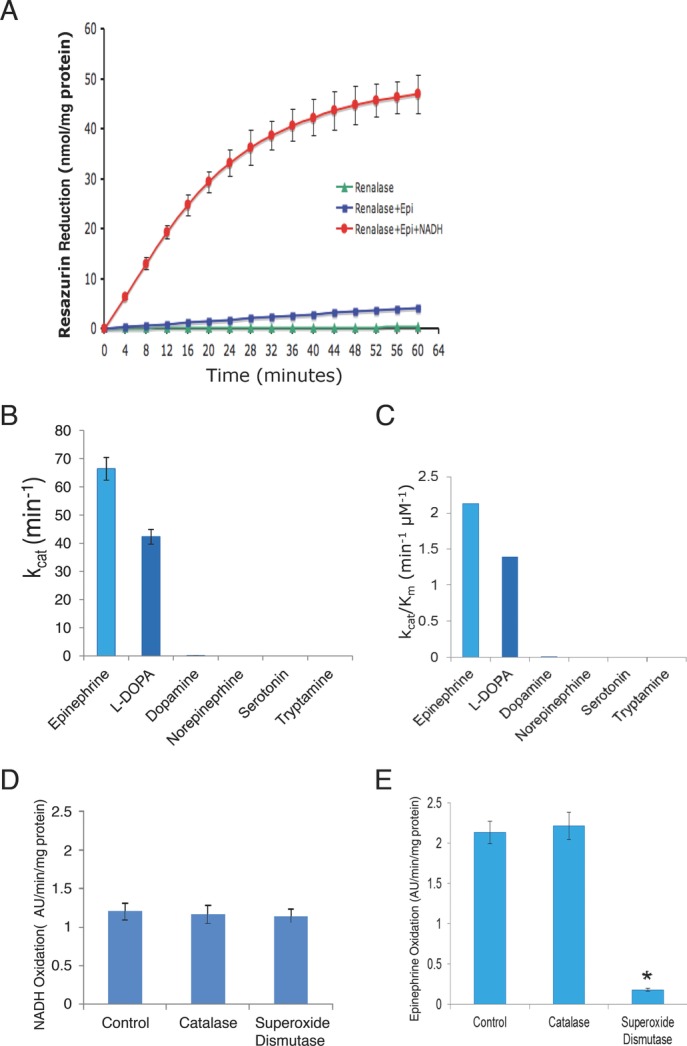
hRenalase1's physiological substrates: epinephrine and l‐DOPA. A, Effect of NADH on renalase's enzymatic activity. Epi indicates 200 μmol/L epinephrine; NADH, 250 μmol/L nicotimamide adenine dinucleotide. Values are mean±SEM; n=3. B, Catalytic rate for selected compounds. Data shown represent steady‐state rate of substrate oxidation by hRenalase1 in 25 mmol/L Tris, pH 7.5, 5 mmol/L NaCl, and 1.5 mmol/L NADH, at 37°C. Dopamine consumption was measured directly by HPLC. Values are mean±SEM; n=4. C, Catalytic efficiency of hRenalase1. n=4. D, NADH oxidation is unaffected by catalase and SOD. E, Dependence of epinephrine oxidation on superoxide anion. Control indicates hRenalase1 in 25 mmol/L Tris, pH 7.5, 5 mmol/L NaCl, and 1.5 mmol/L NADH, at 37°C; Catalase, 100 U catalase added to control reaction; SOD, 100 U SOD added; and AU, change in absorbance at 340 nm. n=5 for each group; Control compared to Superoxide with the Mann‐Whitney test. *P<0.05.

The reduced form of FAD can react with oxygen, as shown in Equation 2, to generate a reactive intermediate such as superoxide anion (O_2_^−^) or hydrogen peroxide (H_2_O_2_) by dismutation of O_2_^−^, which in turn can oxidize epinephrine (Equation 3).

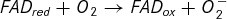
(2)

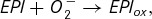
(3)
where O_2_ represents oxygen; O_2_^−^, superoxide anion; EPI, epinephrine; and EPI_ox_, oxidized epinephrine.

In the absence of epinephrine, neither SOD (O_2_^−^ scavenger) nor catalase (H_2_O_2_ scavenger) significantly affects the rate of NADH oxidation (Figure 2D), which suggests that under these conditions the reduced FAD does not react with O_2_. In marked contrast, SOD completely abolishes the epinephrine‐dependent increase in NADH oxidation (Figure 2E), which confirms the catalytic role of O_2_^−^ in epinephrine oxidation and suggests that epinephrine binding stimulates the reaction between reduced FAD and O_2_. Unlike SOD, catalase has no effect on the reaction rate, which indicates that H_2_O_2_ does not play a significant role in renalase‐mediated oxidation of epinephrine (Figure 2E).

To test if epinephrine and l‐DOPA were the only substrates of hRenalase1, we screened 17 246 unique small molecules for their ability to stimulate renalase‐dependent NADH oxidation. In addition to epinephrine and l‐DOPA, 3 additional compounds were identified as potential substrates: dobutamine, isoproterenol, and α‐methyldopa (Figure 3). The first 2 are nonselective β‐adrenergic receptor agonists widely used in clinical practice, whereas the latter is a centrally acting antihypertensive agent. Given that l‐DOPA is synthesized from tyrosine and that hRenalase1's crystal structure revealed significant structural homology not only to MAO‐A but also to an L‐amino acid oxidase,^11^ we also tested if hRenalase1 had amino acid oxidase activity and found none. All the renalase substrates identified to date are aromatic amines, and comparing the chemical structures of renalase substrates to

those of nonsubstrates revealed 2 critical structural features: hydroxylation of the aromatic group at the 3 and 4 positions (isoproterenol versus metaproterenol), which is absolutely required, and methylation of the amine group (epinephrine versus norepinephrine), which can be partially compensated by carboxylation at the α position (l‐DOPA and methyldopa versus dopamine). (Figure 3).

**Figure:**
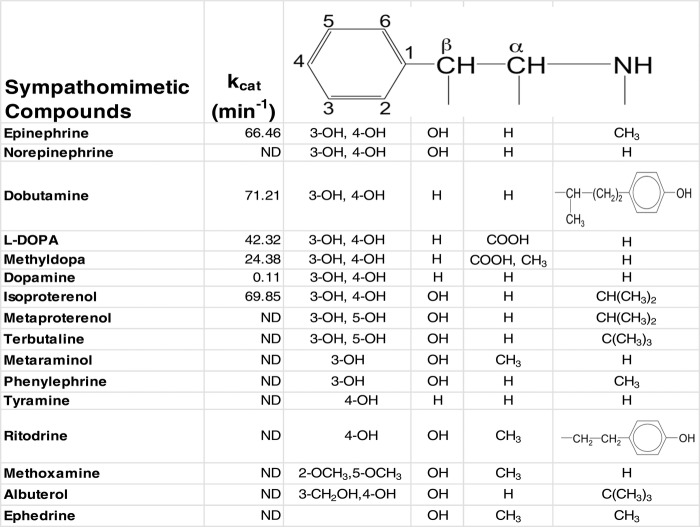
Chemical structure of sympathomimetic compounds metabolized by renalase

### Mechanism of Action of Renalase

Renalase metabolizes epinephrine in vitro, and its deficiency is associated with increased BP and a 3‐fold elevation in plasma epinephrine,^3^ which suggests that its enzymatic activity in vivo is identical to that shown in vitro. To test if renalase's hypotensive effect is linked to its effect on epinephrine metabolism, the protein was administered to renalase‐knockout mice by subcutaneous injection, and plasma epinephrine concentration was measured 24 hours later. Compared to control buffer, hRenalase1 administration led to a 2.56‐fold reduction in plasma epinephrine at 24 hours (Figure 4A). To further test if renalase's hypotensive effect correlates with its enzymatic activity, we generated single–amino acid mutations that decreased its enzymatic activity to varying degrees, and we compared the hypotensive effect of the mutants to that of hRenalase1. The kinetic properties of 4 cysteine‐to‐alanine mutants are compared to those of hRenalase1 in Table 2. The catalytic efficiency (k_cat_/K_m_) of C47A, C54A, C220A, and C327A is reduced by 20%, 95%, 95%, and 94%, respectively. These results also indicate that the enzymatic activity of the sample is accounted for by recombinant hRenalase1 and is not due to contaminating *E coli* enzymes. The hemodynamic effects of hRenlase1, C47A, C54A, C220A, and C327A, administered intravenously to anesthetized mice, also are shown in Table 2. A strong correlation was found between Renalase's catalytic efficiency and its capacity to decrease BP (*r*=0.90, *P*<0.05) (Figure 4B and 4C).

**Table 2.** Kinetic Properties of 4 Cysteine‐to‐Alanine Mutants Compared to Those of hRenalase1

Renalase	k_cat_/K_m_, min^−1^ μmol/L^−1^	Mean ΔBP, mm Hg	Δ Heart Rate, bpm	n
hRenalase1	0.126	−27.6±1.9	−108.9±16.8	12
C47A	0.098	−17.8±4.9	−11.6±17.7	6
C54A	0.006	−10.5±0.33	−8.5±13.0	6
C220A	0.006	−5.3±1.2	6.4±6.2	7
C327A	0.007	−3.8±0.8	1.0±7.4	6

**Figure:**
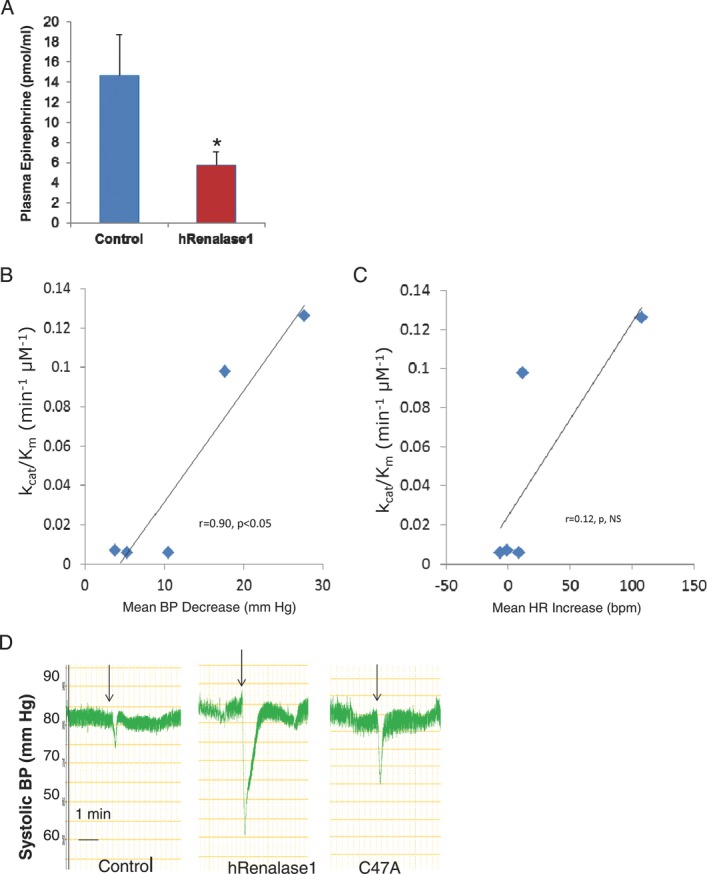
Mechanism of action of renalase. A, Effect of hRenalase1 on plasma epinephrine. Buffer (Control) or 1.5 mg/kg of recombinant renalase (hRenalase1) was administered by a single subcutaneous injection to knockout mice at 8 wk of age, and plasma epinephrine concentrations were measured 24 h later in both groups by HPLC. n=8 for each group. Control compared to hRenalase1 with the Mann‐Whitney test. *P<0.05. B, Correlation between the catalytic efficiency of hRenalase1 and the cysteine to alanine mutants and their effect on BP. Line indicates linear regression; r, correlation coefficient. C, Correlation between the catalytic efficiency of hRenalase1 and the cysteine to alanine mutants and their effect on heart rate. HR indicates heart rate; bpm, beats per minute; line, linear regression; and r, Spearman correlation coefficient. D, Acute effect of WT and C47A on BP. Arrow indicates intravenous injection of buffer (control) or 1 mg/kg of hRenalase1, or 1 mg/kg C47a mutant in anesthetized mice; representative experiment.

Although intravenously administered hRenalase1 significantly decreases BP, the effect is short lived (Figure 4D). We wondered if the short duration of action was due to the pharmacokinetics of intravenous renalase, with rapid inactivation, or excretion by the kidney. We therefore tested whether altering the pharmacokinetics of hRenalase1 by administering it subcutaneously would affect the duration of its hypotensive effect. Although administration of buffer did not affect BP in 5/6 Nx rats (Figure 5A), a single dose of recombinant renalase (1.3 mg/kg) administered subcutaneously decreased both systolic and diastolic BP (Figure 5B). The hypotensive effect of renalase was similar to that of 5 mg/kg enalapril (Figure 5C). Renalase administration decreased BP without any significant effect on heart rate: Mean differences in heart rate compared to control at 12, 24, and 48 hours for renalase were −8.4±5.74, −8.5±6.96, and 5.9±4.82 bpm, respectively. In spontaneously hypertensive stroke‐prone (SHRSP) rats, a single injection of recombinant renalase (1.3 mg/kg) or administration of enalapril (1 mg/kg) decreased both systolic and diastolic BP 12 hours after treatment by ≍7 mm Hg. Although this change in BP is likely to be physiologically relevant, it did not achieve statistical significance in either group (n=4 each). These data indicate that the subcutaneous administration of hRenalase1 is associated with a sustained fall in BP in the 5/6 Nx rat model.

**Figure:**
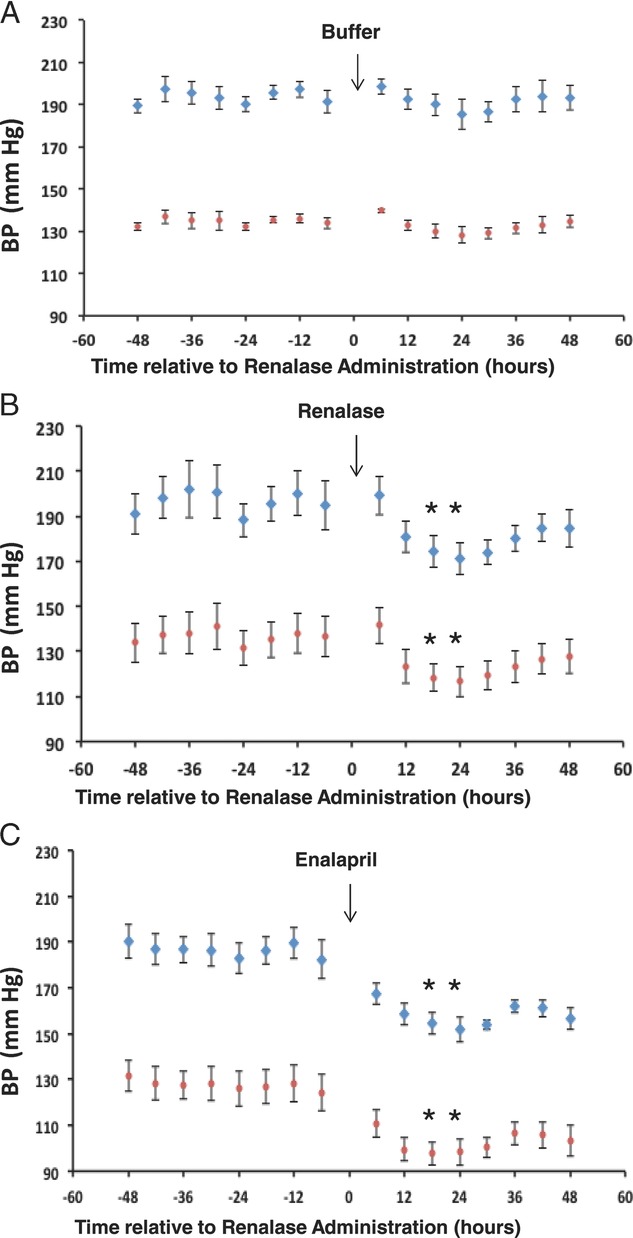
Effect of hRenalase1 on BP in rat models of chronic kidney disease (5/6 Nx). BP was recorded for 2 days before and after treatment. Each data point represents the average of systolic (blue) or diastolic (red) BP values collected every 5 min for 6 h in 4 animals. The standard errors of the means are shown. A, Buffer control in 5/6 Nx rats. Arrow indicates buffer administration. Friedman test revealed no statistical significance. B, Single subcutaneous injection of recombinant renalase in 5/6 Nx rats. Arrow indicates renalase administration. Friedman test revealed statistical significance; Dunn's test was used for pairwise comparisons; *P<0.05. C, Enalapril (5 mg/kg) provided in drinking water for 24 hours to 5/6 Nx rats. Arrow indicates enalapril administration. Friedman test revealed statistical significance; Dunn's test was used for pairwise comparisons; *P<0.05.

## Discussion

Among the circulating catecholamines, hRenalase1 preferentially metabolizes epinephrine (k_cat_=66.46). It metabolizes dopamine at an extremely low rate (k_cat_=0.11), has no measurable activity against norepinephrine, and is, therefore, unlikely to directly regulate norepinephrine and dopamine levels in vivo. Its kinetic parameters for epinephrine compare favorably to those of MAO‐A and MAO‐B, with a higher V_max_ and lower K_m_ than both MAO‐A and the MAO‐B, which suggests that it could significantly contribute to overall epinephrine metabolism in vivo.^16^ Renalase's rather narrow substrate specificity sets it apart from all known amine oxidases. Additionally, to metabolize epinephrine, it uses a mechanism that is different than that used by the MAOs. Indeed, unlike MAOs, it uses NAD(P)H as a cofactor and reacts with oxygen to generate superoxide anion. This reaction scheme is similar to that of flavoprotein monooxygenases, a diverse class of enzymes that can catalyze a wide variety of oxidation reactions, including amine oxidation.^17^ Renalase resembles Class A flavoprotein monooxygenases, by virtue of being the product of a single gene, having a single FAD/NAD(P)H (Rossmann fold)–binding domain, using FAD and NAD(P)H as cofactors, and reacting with oxygen to generate superoxide anion.^17^ These enzymes have been characterized best in bacteria and are usually involved in the degradation of aromatic compounds by para‐ or ortho‐hydroxylation of the benzene ring. Para‐hydroxybenzoate hydroxylase (4‐hydroxybenzoate 3‐monooxygenase), which hydroxylates 4‐hydroxybenzoic acid at the 3 position, is the prototypic enzyme of this subclass. Although 4‐hydroxybenzoate 3‐monooxygenase and hRenalase1 are not homologous at the amino acid level, they do share the p‐hydroxybenzoate hydroxylase fold topology.^11^

Deletion of the renalase gene in mice is associated with hypertension and elevated plasma catecholamines.^3^ Although the largest increase is seen in plasma epinephrine (3‐fold), both dopamine and norepinephrine are also increased, by 1‐ and 0.5‐fold, respectively. What could account for this observation if epinephrine is renalase's main physiological substrate? With regard to norepinephrine, in vitro and in vivo studies indicate that epinephrine stimulates presynaptic β_2_‐adrenergic receptors and facilitates the secretion of norepinephrine from vasoconstrictor nerves.^18,19,20^ It is, therefore, possible that the renalase deficiency leads to a rise in plasma epinephrine, which subsequently stimulates the release of norepinephrine. A different mechanism could account for the increase in plasma dopamine in the renalase‐knockout mouse. In these animals, it is possible that the renal dopaminergic system is upregulated in an attempt to maintain sodium balance and mitigate the rise in BP associated with renalase deficiency. In addition, because hRenalase1 metabolizes l‐DOPA (the other natural substrate identified), plasma levels would be expected to rise in the renalase knockout, and this could partly account for the stimulation of renal dopamine synthesis.

Most importantly, the data indicate that renalase's hypotensive effect is mediated by its ability to metabolize circulating epinephrine, its principal physiological substrate. It is noteworthy that the catalytic efficiencies of C54A, C220A, and C327A are similarly decreased, whereas C54A's effect on BP is greater than that of C220A and C327A. Catalytic efficiency (k_cat_/K_m_) is shown in Table 2 because the mutations affect both k_cat_ and K_m_. The C54A mutation does not significantly affect k_cat_ but markedly decreases substrate affinity (K_m_). This is in contrast to the C220A and C327A mutations, which affect both k_cat_ and K_m_. One possibility is that changes in both K_m_ and k_cat_ modulate the observed hypotensive effect and that k_cat_ plays a stronger role than K_m_ in determining the in vivo response.

An extensive search for physiological substrates revealed only 1 additional substrate, namely l‐DOPA, the main precursor of catecholamines. It should be noted that small chemical screens have inherent limitations relating to the finite numbers of molecules that can be reasonably tested, as well as the fact that novel (unknown) compounds would not be identified. In the present study, >17 000 compounds were screened by using libraries that contained a large number of endogenous compounds. Additionally, on the basis of information obtained from the crystal structure of renalase, we screened additional compounds not present in the chemical libraries. In the end, only catecholamines and catecholamine‐like molecules behaved as substrates of renalase. We have no evidence that the recombinant renalase that is administered is taken up by cells; therefore, we postulate that it is acting either within the intravascular compartment or in the interstitial fluid, where it could diffuse into nerve terminals and modulate catecholamine levels. Renalase is expressed in the spinal cord and in peripheral nerves and could modulate catecholamine concentration at the neuromuscular junction.^2^

In the vasculature, the data suggest that recombinant renalase would acutely decrease epinephrine levels. It is clear that renalase has intrinsic NADH oxidase activity in the absence of substrates and that its V_max_ and K_m_ for NADH are such that it could contribute to the intracellular and extracelular NAD+ pool and affect cell signaling pathways. Recombinant hRenalase1 administered subcutaneously has a potent hypotensive effect in 5/6 Nx and SHRSP rats. Systolic and diastolic ambulatory BP is significantly decreased for up to 12 and 48 hours in SHRSP and 5/6 Nx rats, respectively. The potency and duration of renalase's hypotensive effect are magnified in chronic kidney disease. This most likely is explained by a decrease in urinary excretion that results in higher plasma levels in chronic kidney disease. A single subcutaneous injection of 1.3 mg/kg hRenalse1 is as effective in decreasing BP as is 5 mg/kg enalapril (10‐fold recommended dose) administered orally over 24 hours, which suggests that renalase has a potent hypotensive effect. It is noteworthy that, unlike β‐blockers and calcium channel blockers, recombinant renalase given subcutaneously decreases BP without any significant change in heart rate.

In summary, hRenalase1 represents a new class of epinephrine‐metabolizing enzyme that is distinct from MAO‐A and MAO‐B with regard to cellular distribution (soluble, secreted versus membrane bound), FAD binding (noncovalent versus covalent), active conformation (monomeric versus dimeric), and cofactor requirement [NAD(P)H]. The data suggest that hRenalase1 modulates BP through its action on circulating epinephrine and represents a novel therapeutic target for the treatment of hypertension.
